# Expression of IL-1α correlates with distant metastasis in patients with head and neck squamous cell carcinoma

**DOI:** 10.18632/oncotarget.6054

**Published:** 2015-10-09

**Authors:** Xavier León, Carolina Bothe, Jacinto García, Matilde Parreño, Sonia Alcolea, Miquel Quer, Luis Vila, Mercedes Camacho

**Affiliations:** ^1^ Department of Otorhinolaryngology, Hospital de la Santa Creu i Sant Pau and Universitat Autònoma de Barcelona, Barcelona, Spain; ^2^ Laboratory of Angiology, Vascular Biology and Inflammation, Institute of Biomedical Research (IIB Sant Pau) and Universitat Autònoma de Barcelona, Barcelona, Spain; ^3^ Laboratory of Translational Molecular Oncology, Institute of Biomedical Research (IIB Sant Pau) and Universitat Autònoma de Barcelona, Barcelona, Spain; ^4^ Centro de Investigación Biomédica en Red de Bioingeniería, Biomateriales y Nanomedicina (CIBER-BBN), Madrid, Spain

**Keywords:** head and neck cancer, IL-1α, metastasis, prognosis

## Abstract

The presence of IL-1 in human cancers is associated with aggressive tumor biology but its prognostic value is unknown. We studied whether IL-1α expression is a prognostic marker of distant metastasis in patients with head and neck squamous cell carcinoma (HNSCC). IL-1α mRNA and protein levels were determined in tumor samples and cancer cell lines using RT-PCR and ELISA. The effects of constitutive IL-1α expression by tumor lines were characterized. IL-1α mRNA and protein secretion were higher in tumor samples from patients who later developed distant metastasis than in patients who did not. By using distant metastasis as a dependent variable, patients were classified into two categories of IL-1α transcript-levels. The high-IL-1α group had a significantly lower five-year distant metastasis-free survival than the low-IL-1α group [70.0% (CI 95%: 55.9-84.1%) *vs* 94.7% (CI 95%:90.2-99.2%)]. When IL-1α transcript-levels were combined with clinical factors related to tumor metastasis, the predictive power of the model increased significantly. Additionally, transcript levels of IL-1α correlated significantly with those of the IL-1 family genes and genes related to the metastatic process. IL-1 treatment of microvascular endothelial cells increased adhesion of HNSCC cells but no differences were found based on constitutive IL-1α expression by tumor cells. Nevertheless, IL-1α produced by tumor cells effectively increased their transmigration across the endothelium. We found a significant relationship between IL-1α expression and development of distant metastasis in HNSCC patients. IL-1α expression could help to define a subset of patients at high risk of distant metastasis who could benefit from adjuvant treatment.

## INTRODUCTION

Loco-regional control of head and neck squamous cell carcinoma (HNSCC) has improved in the last decades thanks to more aggressive surgical techniques, improvements in radiotherapy, and the development of new antitumoral drugs. Paradoxically, these advances have not translated into a concomitant increase in survival. The appearance of distant metastasis is one of the most relevant factors responsible for this divergence [1].

Metastatic spread is a complex sequential process which depends on the biology of the tumor. Tumor metastasis is a sequential multi-step process requiring detachment of individual tumor cells from the adjacent tumor and stromal cells, the invasion and progression through the extracellular matrix, the intravasation into lymphatic or vascular vessels, survival in regional or systemic circulation, and implantation and proliferation into regional neck nodes or distant tissues [2, 3]. All these steps are tightly regulated by cell-associated and soluble mediators. Tumors secrete soluble factors such as growth factors, cytokines and chemokines with autocrine and paracrine biological activities. These factors not only exert autocrine actions but also interact with the surrounding stromal and endothelial cells, exerting relevant biological effects. One of these effects is the promotion of metastasis, an effect which has been related with several molecules expression, such as desmoplakin [4], E-cadherin and vimentin [5, 6], αB-crystallin (HspB5) [7] or IL-α [8].

IL-1 is a pleiotropic pro-inflammatory cytokine that exhibits biological activities supporting its role in tumor growth and metastasis. The IL-1 family includes three members that bind to the same receptor. Two of the members are active, IL-1α and IL-1β, and the third member is an antagonist, IL-1Ra. When IL-1Ra binds to the IL-1 receptor, no signal is transduced and there is no biological response. Although IL-1α and IL-1β are coded by different genes, their biological activities are identical. Two receptors of the immunoglobulin superfamily (IL-1R1 and IL-1R2) selectively bind IL-1. IL-1R1 is biologically active and it is ubiquitously expressed, while IL-1R2 acts as an IL-1 scavenger. Following binding of IL-1 to IL-1RI the IL-1 receptor accessory protein (IL-1RaP) is recruited to form a protein complex. This heterodimeric complex triggers the IL-1 signaling pathway that ultimately leads to the activation of a number of transcription factors [9].

IL-1 induces several pro-metastatic genes, such as matrix metalloproteinases and endothelial adhesion molecules, and pro-angiogenic factors such as VEGF, chemokines, prostaglandin (PG) E_2_, growth factors, and TGFβ [10]. Expression of IL-1 is high in some tumors [11], including HNSCC [12-14]. Studies with experimental models of melanoma [15], pancreatic carcinoma [16, 17], lung carcinoma and colon cancers [18] have shown that local production of IL-1 influences metastatic capacity and tumour growth [8, 19]. Moreover, tumors that produce high levels of IL-1 have been associated with a poor prognosis [11]. IL-1 induces angiogenesis *in vivo* and *in vitro* [20].

Data concerning the role of IL-1 expression in the appearance of distant metastasis have not been evaluated in HNSCC patients. The aim of the present study was to assess IL-1 expression in pre-treatment biopsy samples of HNSCC patients, and to evaluate the prognostic capacity of IL-1 levels in the prediction of the appearance of distant metastasis.

## RESULTS

### IL-1α transcript levels are associated with the presence of distant metastasis

**Figure 1:**
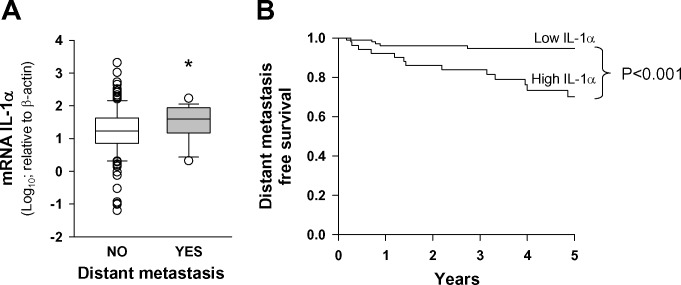
IL-1α expression is associated with the presence of distant metastasis **A.** Box plot graph of IL-1α transcript levels relative to β-actin (x1000) in HNSCC samples as a function of appearance of distant metastasis; metastasis (*n* = 18) and no metastasis (*n* = 136); *, *p* < 0.05; when compared with the no-metastasis group of patients. **B.** Five-year distant metastasis-free survival according to the category of the IL-1α mRNA levels; low IL-1α *n* = 102 and high IL-1α *n* = 52.

Table 1 shows the characteristics of the patients included in the study. The median follow-up time of patients was 4.8 years (range, 2.3-10 years). The median follow-up time of patients was 4.8 years (range, 2.3-10 years). Local failure of the tumor occurred in 46 (29.9%) patients during the follow-up period, regional failure was detected in 23 (14.9%) patients, and distant metastasis in 18 (11.7%) patients. We examined the expression of IL-1α in tumoral samples from HNSCC patients with and without distant metastasis by quantitative RT-PCR (Figure 1A). Patients with distant metastasis showed significantly higher levels of IL-1α than those without metastasis. Using appearance of distant metastasis as the dependent variable, CART analysis classified patients into two categories, low (*n* = 102, 66.2%) and high (*n* = 52, 33.8%) expression level of IL-1α. The five-year distant metastasis-free survival was statistically significant between the two groups (*p* < 0.001), being 70.0% (CI 95%: 55.9-84.1%) for patients with an IL-1α high-expression level and 94.7% (CI 95%:90.2-99.2%) for patients with an IL-1α low-expression level. Figure 1B shows the distant metastasis-free survival curves corresponding to the CART classification. Table 2 shows the results of the multivariate study considering the appearance of distant metastasis as the dependent variable. The expression level of IL-1α was the only variable that was significantly related to the appearance of distant metastasis. Considering patients with a low-level expression of IL-1α as the reference category, patients with high expression of IL-1α had a 5.3-fold higher risk of appearance of distant metastasis (CI 95%: 1.8-15.8).

**Table 1:** Characteristics of the patients included in the study

Characteristics	N° (%)
No Patients	154
Age (years) Median Range	62.5 38.1-92.3
Sex Male Female	139 (90.3%) 15 (9.7%)
Tumor location Oral cavity/oropharynx Larynx/hypopharynx	65 (42.2%) 89 (57.8%)
T category cT1-2 cT3-4	80 (51.9%) 74 (48.1%)
N category cN0 cN+	90 (58.4%) 64 (41.6%)
Tumor differentiation Well differentiated Moderately differentiated Poorly differentiated	9 (5.8%) 128 (83.1%) 17 (11.0%)
Treatment Surgery ± radiotherapy (Chemo)-radiotherapy	43 (27.9%) 111 (72.1%)

**Table 2:** Results of the multivariate study considering the appearance of distant metastasis as the dependent variable (HR: hazard ratio)

Characteristics	HR	CI 95% HR	P
Location Oral cavity/oropharynx Larynx/hypopharynx	1 1.56	0.58-4.19	0.37
Local extension cT1-2 cT3-4	1 0.66	0.23-1.88	0.44
Regional extension cN0 cN+	1 2.33	0.82-6.60	0.11
Treatment Surgery ± radiotherapy (Chemo)-radiotherapy	1 0.53	0.20-1.44	0.21
IL-1α Low High	1 5.35	1.81-15.84	0.002

We then analyzed the relationship between the risk of appearance of distant metastasis and the category of IL-1α expression in the group of 99 patients with loco-regional control of the disease. In this subset of patients, the expression level of IL-1α showed the same relationship with the development of distant metastasis. The five-year distant metastasis free survival was 75.2% (CI 95%:59.4-91.0%) for patients with a high-IL-1α and 94.3% (CI 95%:88.9-99.7%) for patients with a low-IL-1α; the difference was statistically significant (*p* = 0.01).

We defined a cohort of patients with a high risk of appearance of distant metastasis based on clinical variables. These variables included locally advanced tumors (T4), advanced regional spread (N2-3), and extracapsular spread. Patients that met one or more of these criteria constituted the high risk group (*n* = 60), while the low risk group did not present any of the criteria (*n* = 94). The low risk group showed a 93.9% (CI 95%: 88.6-99.2%) five-year distant-metastasis-free survival while the high risk group dropped to 71.9% (CI 95%: 58.2-85.6%); the difference was statistically significant (*p* = 0.001). IL-1α expression in the clinical high risk group (*n* = 60) retained prognostic power. Five-year distant-metastasis-free survival for patients with low-IL-1α expression (*n* = 33) was 93.5% (IC 95%: 84.9-100%), dropping to 49.1% (IC 95%: 26.6-71.6%) for patients with high-IL-1α expression (*n* = 27, *p* = 0.002).

Information regarding HPV status was available for 40 of the 49 patients with an oropharyngeal carcinoma. Only 20% (*n* = 8) of oropharyngeal tumors were HPV positive (7, HPV-16 and 1 HPV-51). There were no significant differences regarding IL-1α expression as a function of HPV status (*p* = 0.377).

### Secretion of IL-1α by tumor samples was significantly higher in patients that developed distant metastasis

**Figure 2:**
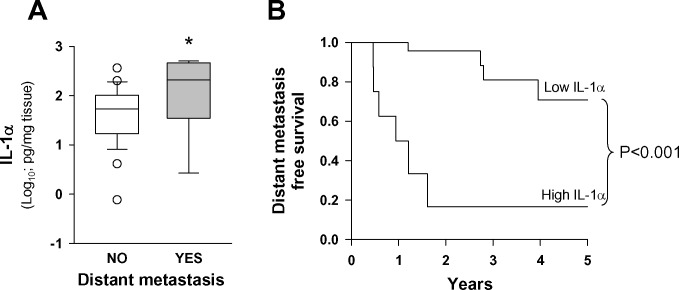
Levels of IL-1α secreted by tumor samples were higher in patients that developed distant metastasis **A.** Release of IL-1α protein by HNSCC samples as a function of appearance of distant metastasis; tissue fragments were incubated for 48 hours in a cell incubator. Thereafter, IL-1α in the media was analyzed; metastasis (*n* = 10) and no metastasis (*n* = 25); *, *p* < 0.05; when compared with the no-metastasis group of patients. **B.** Five-year distant metastasis-free survival according to the category of the IL-1α secreted; low IL-1α *n* = 27 and high IL-1α *n* = 8.

We analyzed IL-1α production *ex vivo* by tumor samples from 35 patients (28.6%) included in the study, 10 of whom developed distant metastasis during the follow-up period. The results in figure 2A show that the protein levels of IL-1α secreted by tumor samples of patients that developed distant metastasis were significantly higher. When we applied the CART method using the development of distant metastasis as the dependent variable, the resulting tree distributed the patients into two groups according to IL-1α concentration in the secretome. The high IL-1α concentration group (*n* = 8) showed a significantly higher risk of distant metastasis than the low IL-1α concentration group (*n* = 27). Figure 2B shows the metastasis-free survival curve obtained with the IL-1α data from the secretome analysis.

### Expression of IL-1α in HNSCC tumor samples correlates with transcript levels of genes related with the metastatic process

**Figure 3:**
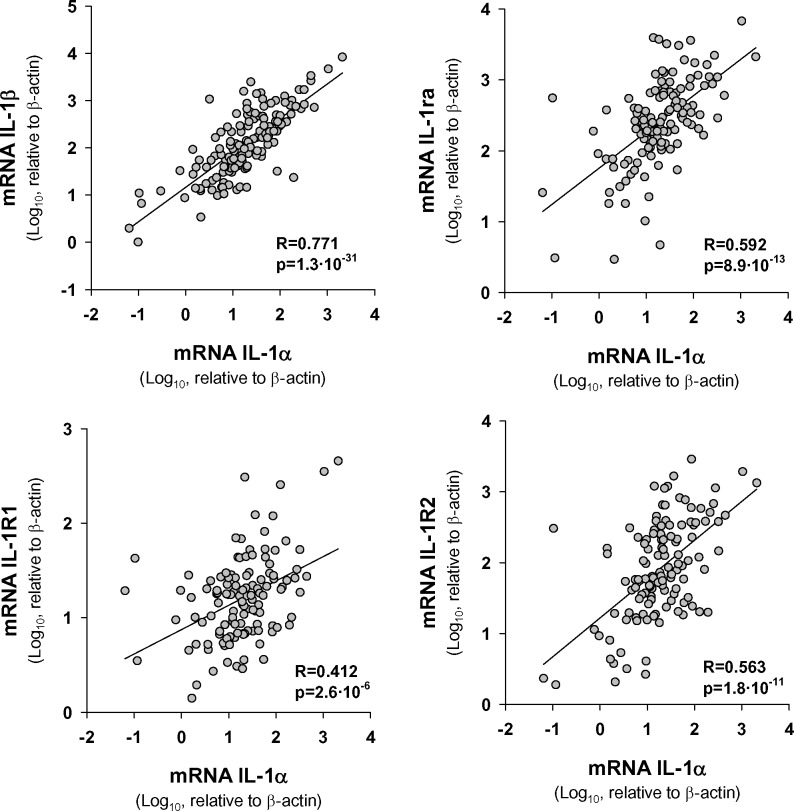
Statistical correlation between transcript levels of IL-1α and those of IL1 signaling pathway genes The Pearson Product Moment Correlation after logarithmic transformation of data was applied; *n* = 154.

We studied the correlation between the expression of IL-1α and the expression of other related genes of the IL-1 family. Results in Figure 3 show a highly significant statistical association between IL-1α and either IL-1β or IL-1Ra. Moreover, we observed that the variation in levels of IL-1α correlated with that of IL-1 receptors, particularly with IL-1R2.

**Figure 4:**
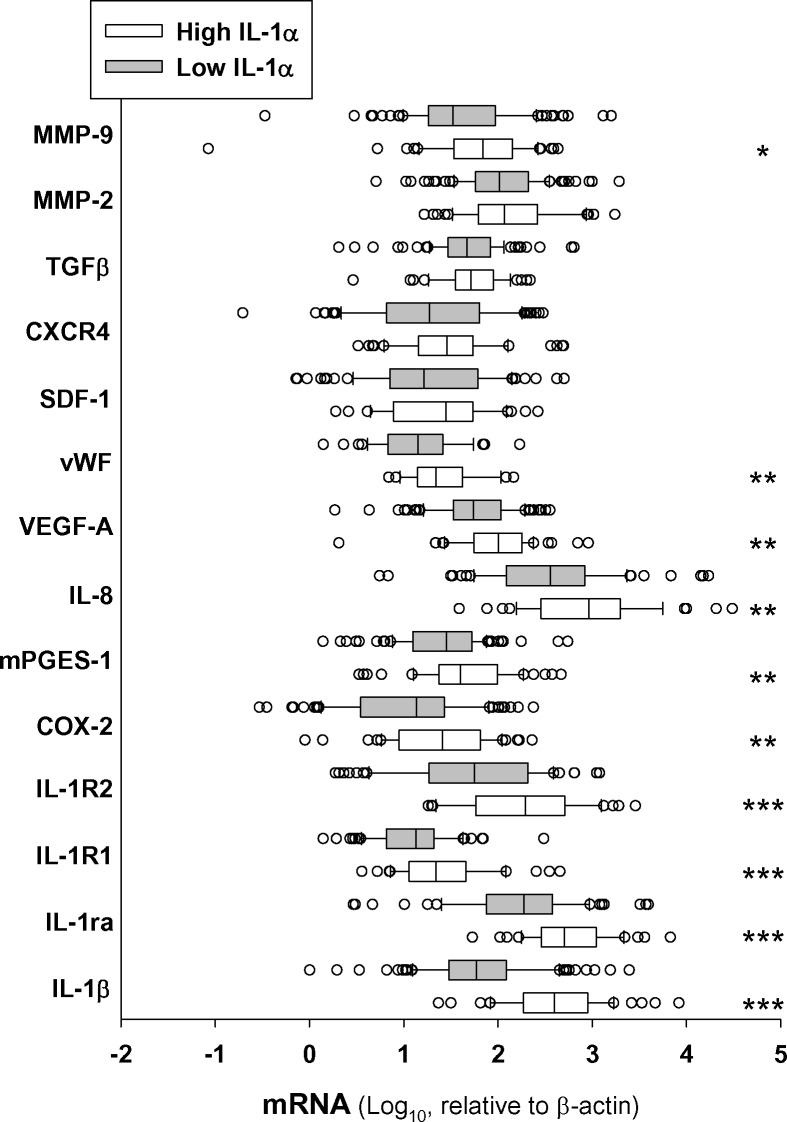
Expression of IL-1α in HNSCC tumor samples correlates with transcript levels of genes related with the metastatic process Box plot of mRNA transcript levels of several genes related or regulated by IL-1 in tumor samples stratified by low and high levels of IL-1α. Low IL-1α *n* = 102 and high IL-1α *n* = 52; *, *p* < 0.05; **, *p* < 0.01 and ***, *p* < 0.001 when compared with low-IL-1α.

We next determined transcript levels of some genes related with the metastatic process that it is known to be regulated by IL-1. Results in figure 4 indicate that most of these genes were enhanced in the high IL-1α group. Interestingly, the most enhanced expression, besides the IL-1 family genes, were the genes involved in angiogenesis, such as MMP-9, PGE_2_ biosynthetic machinery (COX-2 and mPGES-1), VEGF and IL-8. This was consistent with a significant high expression of the endothelial cell marker von Willebrand Factor (vWF) in the high IL-1α group of patients. In contrast, we found no statistically significant differences between patients stratified by the IL-1α expression in other IL-1α-related pathways, such as the chemokine for stem cells SDF-1 or SDF-1-receptor (CXCR4), the matrix degrading enzyme MMP-2, or the epithelial-mesenchymal transition promoter TGFβ (Figure 4).

### Expression of IL-1α increases trans-endothelial cell migration but not affects adhesion of HNSCC cells lines to MVEC

**Figure 5:**
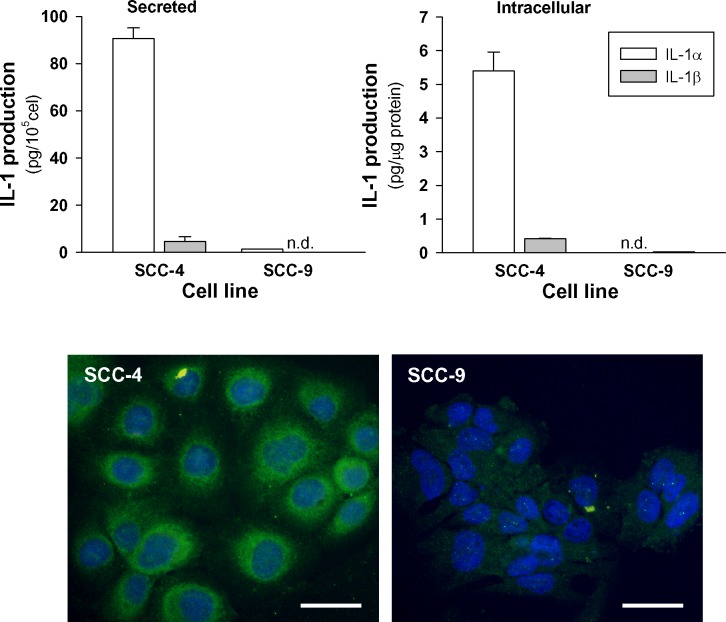
Secreted and intracellular levels of IL-1α and IL-1β by HNSCC cell lines Cells were incubated for 48 hours in 1% FBS. Thereafter, IL-1α and IL-1β were analyzed in the media and in the cell lysates (*n* = 3). Representative immunofluorescent staining for IL-1α is also shown (bars 50μm).

Seven human HNSCC cell lines (CAL-27, FaDu, SCC-4, SCC-9, SCC-22a, SCC-22b and SCC-25) were studied for the expression of IL-1α in terms of mRNA and protein to find two cell lines expressing high and low levels of the cytokine. The cytokine protein was measured simultaneously in both the culture supernatants (for secreted cytokine) and the lysates made from the remaining cells (for cell associated cytokine). All HNSCC cell lines assayed, except SSC-9, expressed IL-1α but the levels varied from one line to another (data not shown). We chose two tongue carcinoma cell lines SCC-4 and SCC-9. SCC-4 produced high amounts of IL-1α and, to a much lesser extent IL-1β. In SCC-9, levels of both forms of IL-1 were almost undetectable (Figure 5).

**Figure 6:**
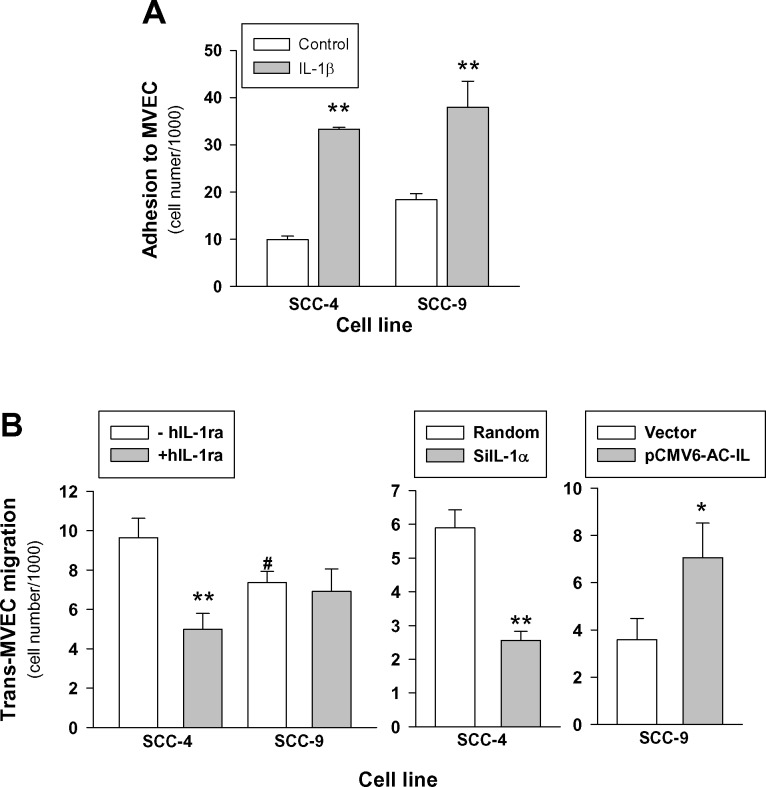
Expression of IL-1α influences trans-endothelial cell migration but not adhesion in HNSCC cells lines **A.**- Effect of IL-1 on the HSCC cells adhesion to MVEC. MVEC cultured in 12-well cell culture plates were treated or not with 10 U/mL IL-1β for 24 hours. MVEC were washed and 1*×*10^6^ fluorescent labeled tumoral cells were added and incubated at 37°C for 30 minutes. Adherent cells were solubilized and fluorescence was measured; SCC-4 *n* = 3; SCC-9 *n* = 7; mean±SEM; **, *p* < 0.01 when compared with controls. **B.**- Effect of the presence of IL-1Ra (left panel; *n* = 7; **, *p* < 0.01 when compared with cells in the absence of IL-1Ra), IL-1α knockdown (middle panel; *n* = 6; *, *p* < 0.05 when compared with random) and IL-1α overexpression (right panel; *n* = 5; *, *p* < 0.05 when compared with empty vector) on the trans-MVEC migration of HNSCC cell lines. MVEC were plated onto 24-well cell culture inserts 8-μm. Once confluence was reached, 2.5*×*10^5^ fluorescent labeled tumoral cells were added to the upper well of the inserts and allowed to transmigrate through the endothelium for 24 h. Migratory cells were lysed and fluorescence was measured. Left panel in the absence (-hIL-1ra) or in the presence (+ hIL-1ra) of 250 ng/mL recombinant human IL-1Ra; middle panel SCC-4 transfected with siRNA targeting IL-1α (siIL-1α), negative control siRNA (Random); right panel SCC-9 transfected with plasmid encoding full-length IL-1α pCMV6-IL-1α (pCMV6-AC-IL) or Precision Shuttle mammalian vector pCMV6-AC (Vector).

We then examined the influence of IL-1α expression on the ability of tumor cells to adhere to MVEC and MVEC previously treated with 10U/mL of human recombinant IL-1β for 24 hours. The results in Figure 6 show that both SCC-4 and SCC-9 cells adhere to MVEC and treatment of MVEC with IL-1β significantly increased the adhesion of tumor cells, but no differences were found between the two cell lines. Afterwards, we tested the ability of these cell lines to migrate across a MVEC monolayer *in vitro*. Results depicted in the left panel of Figure 6B show that both cell lines were able to migrate across the MVEC barrier, and although the transmigration of SCC-4 was higher in terms of the mean, the difference between the two cell lines failed to achieve statistical significance (*p* = 0.08). Nevertheless, the presence of human recombinant IL-1Ra significantly reduced transmigration of SCC-4, whereas it failed to block SCC-9 transmigration. While the above observation suggested a correlation between IL-1α expression and tumor cell transmigration, we further tested the effect of IL-1α knockdown and overexpression on the trans-MVEC migration. We found that silencing of IL-1α in SCC-4 significantly reduced transmigration across MVEC and overexpression of IL-1α in SCC-9 resulted in a significant increase of cell transmigration (Figure 6B). However, neither IL-1α knockdown nor IL-α overexpression significantly affected tumoral cells adhesion to matrix extracellular proteins (data no shown).

## DISCUSSION

Our study in a large cohort of HNSCC patients provide evidence that high levels of IL-1α expression are associated with a higher risk of distant metastasis, in terms of both local transcript in the primary tumor and *ex vivo* protein production by primary tumor samples.

The appearance of distant metastasis is a major cause of death in head and neck cancer patients. The rate of clinical progression, including the risk of metastasis and relapse, can be estimated on the basis of clinical information such as tumor location, lymph node status at diagnosis and extracapsular spread of the lymph nodes [21-23]. However, only a few studies have related biomarker expression with the risk of distant metastasis in HNSCC as, for example, down-regulation of desmoplakin [4], cytokeratin 19 [24] or E-cadherin [5, 6], and over-expression of αB-crystallin [7].

Previous reports have emphasized the increased risk of distant metastasis in patients with locoregional failure after initial treatment [25, 26]. This increased risk may be due to unsuccessful treatment or a sign of tumor aggressivity. Furthermore, patients who achieve locoregional control of the primary tumor may also later develop metastasis if there is subclinical metastatic spread at the time of locoregional treatment. Our results showed that in both clinical settings, with or without locoregional relapse, expression of IL-1α has significant prognostic power.

In the tumor milieu, IL-1 induces expression of various metastatic mediators such as MMP, VEGF, IL-8, IL-6, TNFα, and TGFβ (reviewed in 9,13,30,31). Our correlation study showed that variations in tumor IL-1α transcript levels were associated not only with variations of IL-1 family genes, but also with several genes related to neovascularisation. This suggests that IL-1α stimulates vascularization, thus facilitating tumor cell transmigration. The fact that IL-1 induces angiogenesis *in vitro*, mainly promoting PGE_2_ biosynthesis, supports this suggestion [20]. We previously showed that HNSCC cell supernatant induced COX-2 and mPGES-1 in fibroblasts and MVEC mainly by means of IL-1 receptor activation, increasing PGE_2_ production and activity [14, 27]. Also, IL-1α released by tumor cells stimulates the proliferation of carcinoma-associated fibroblasts and increases cytokines secretion from fibroblasts, which in turn promotes cancer progression [8]. Taken together, these findings show that IL-1α is an important factor in the crosstalk between tumoral cells and surrounding stroma and endothelial cells.

The first step in the process of metastatic spread is the binding of tumoral cells to endothelium. IL-1 increases adhesiveness of endothelial cells up-regulating the expression of endothelial adhesion molecules. Here, we show that treatment of MVEC with IL-1 enhanced adhesion of tumoral cells. We also show a correlation between IL-1α expression and tumor cell transmigration. IL-1α produced by tumor cells effectively increased its transmigration across the endothelium. This conclusion is based on the fact that the presence of IL1Ra resulted in a statistically significant decrease in trans-endothelial migration of an IL1-producing cell line tested but it had no effect on non-IL-1-producing cells. In addition, IL-1α knockdown in the IL1α-producing cell line significantly reduced transmigration, while overexpression of IL-1α in the non-IL-1α-producing cell line significantly increased cell transmigration.

However, tumors are heterogeneous population of cells from diverse origins, such as stem cells, stromal cells, endothelial cells and a wide range of immune cells. IL-1 is secreted by tumor cells, but others, particularly immune cells infiltrating tumor, may contribute to the levels of IL-1 in the tumor microenvironment [10, 19, 28, 29]. Further research will be required to relate the degree of infiltrating cells and their activation with the tumor levels of IL-1 and therefore their role in metastasis mediated by IL-1 in HNSCC.

The combination of clinical data with biomarkers such as IL-1α could help us to define a population at high risk for distant metastasis. In this study, patients with a high expression level of IL-1α and clinical high risk prognostic factors showed a 50% risk of distant metastasis in five years. These patients could benefit from adjuvant treatments that help sterilize the micrometastatic disease thereby improving disease-free survival. Nevertheless, some limitations of the study should be taken into account. This is a single institution retrospective study with a limited number of patients. Independent validation studies are required to demonstrate the utility of IL-1α expression as a biomarker for distant metastases in this clinical setting.

In conclusion, our results show a significant relationship between IL-1α expression and the development of distant metastasis in HNSCC patients. The combination of clinical data, such as locoregional extension or extracapsular spread and IL-1α expression could help us to define a group of patients with a high risk of distant metastasis. Adjuvant treatment in this high-risk group could prove useful in terms of disease control and long term survival. Independent validation studies are required to prove the utility of IL-1α expression as a biomarker for distant metastasis in the clinical setting.

## MATERIALS AND METHODS

### Patients

The biopsies used in the present study were obtained from 154 consecutive patients with a pathologically confirmed HNSCC treated at our centre between 2004 and 2010. All the patients were evaluated by the institution's cancer committee, and the decision to treat them with induction chemotherapy, surgery, radiotherapy or chemo-radiotherapy was based on institutional guidelines. The clinical data were obtained from a database which prospectively collects information about the clinical status, tumor characteristics, treatment, and follow-up of all patients treated in our centre since 1985 [30].

Table 1 shows the characteristics of the patients included in the study. All patients had a minimum follow-up for 2 years. The present study was approved by the local ethics committee, and informed consent was obtained from each subject. All procedures were reviewed by the Institutional Review Board at Hospital de la Santa Creu i Sant Pau. The study conforms to the principles outlined in the Declaration of Helsinki.

### Analysis of mRNA levels

Tumor tissue samples obtained from each patient were stabilized by inclusion in RNA-later (Quiagen GmbH, Hilden, Germany) immediately after biopsy, and stored at −80°C until processing. Total RNA was extracted using Trizol (Invitrogen, Carlsbad, CA) following the manufacturer's instructions and RT-PCR was performed as described previously [20]. Relative expression was expressed as transcript /β-actin ratios.

### Determination of IL-1α released by HNSCC tumor samples

IL-1α secretion was analyzed in 35 tumor biopsies. 100-200 mg tissue fragments were incubated in 0.5 mL of DMEM (Biological Industries, Kibbutz Beit Haemek, Israel) for 48 h in a cell incubator. Culture medium was then recovered and kept at −80° C until IL-1α analysis.

### Tumor cell culture

SCC-9 (CRL-1629, tongue carcinoma) and SCC-4 (CRL-1624, tongue carcinoma) were obtained from American Type Culture Collection (ATCC) and cultured as recommended by the supplier.

### Isolation and culture of human microvascular endothelial cells (MVEC)

MVEC were isolated from human adult foreskins as previously described [27, 31]. All experimental studies were performed with Dynabeads CD31-purified MVEC passage 2 to passage 6.

### Analysis of IL-1α and IL-1β proteins

Quantitative analysis of IL-1α and IL-1β in the culture media was performed by specific ELISA following the manufacturer's instructions; IL-1α was from R&D Systems (Minneapolis, MN) and IL-1β was from eBioscience (San Diego, CA).

### Plasmid/siRNA transfection of tumoral cells

Silencer predesigned siRNA targeting IL-1α (ID:s7266) or Silencer™ Negative Control#1 siRNA (ID:4390843) used in knockdown experiments were obtained from Ambion. Plasmid encoding full-length IL-1α pCMV6-IL-1α (SC324639) and the control empty vector pCMV6-AC (PS100020) were purchased from Origene Technologies (Rockville, MD). siRNA and plasmids were transfected using Nucleofector™ technology (Amaxa Inc) with the “Amaxa Cell Line Nucleofector kit V” according to the manufacturer's recommendations [32]. Briefly, 3-5 × 10^6^ log phase cells were suspended in 100 μL of Nucleofector Solution V, mixed with 3 μg of siRNA or plasmid DNA, and electroporated with the program X-001. After electroporation, cells were suspended in 500 μL of pre-warmed cell culture medium and seeded in complete medium for 6 h after which the medium was changed to serum-free medium containing 0.5% BSA. Gene knockdown and overexpression was verified measuring IL-1α in the cell culture supernatants by ELISA.

### Tumoral cells adhesion and trans-endothelial migration assays

Tumoral cells starved overnight in serum-free medium containing 0.5% BSA were harvested and suspended in PBS at a cell density of 1*×*10^6^ cells/mL. 1,1*_*-dioctadecyl-3,3,3*_*,3*_*-tetramethyl-indocarbocyanine perchlorate (Dil, Sigma) in ethanol was added to yield a 10 μg/mL final concentration and incubated for 1 hour at 37°C. After washing twice with serum-free medium, cells were suspended in DMEM containing 0.5% BSA at the adequate cell density.

For the adhesion assay, MVEC cultured in 12-well cell culture plates until confluence was reached were treated or not with IL-1β for 24 hours in MCDB containing 1% SBF. MVEC were washed twice before the addition of 0.5 mL of DMEM with 1% FBS and warmed at 37°C for 5 minutes before the addition of 1*×*10^6^ labeled tumoral cells suspended in 0.5 mL of medium. The cells were then incubated at 37°C for 30 minutes. Thereafter, non-adherent cells were thoroughly washed with PBS and 150 μL of 1% Triton X-100 was added to each well. Fluorescence was measured in a Synergy™ HT Multi-Detection Microplate Reader (Biotek) using a 530/590 filter set. Cell number was determined by running a fluorescent cell dose curve for each assay.

For the trans-endothelial migration assays, MVEC were plated onto 24-well Millicell cell culture inserts 8-μm PET (Millipore, Billerica, MA) and allowed to form a confluent monolayer. Once confluence was reached, the culture medium from the inserts was removed and the inserts were transferred to new wells containing 600 μL of serum-free DMEM 0.5% BSA. 2.5*×*10^5^ of fluorescent labeled tumoral cells in 0.25 mL of medium were added to the upper well of the inserts in the presence or the absence of 250 ng/mL recombinant human IL-1Ra and allowed to transmigrate through the endothelium for 24 h. Thereafter, non-migratory cells were removed by aspirating and inserts were placed into new wells containing 0.5% trypsin in PBS. Cells were completely dislodged from the underside of the membrane by gentle tilting. They were then centrifuged and lysed. Fluorescence was measured as for adhesion assays.

### Statistical analysis

When data fitted a normal distribution, the Student *t*-test was used to compare groups. The Mann-Whitney Rank Sum Test was used when normality failed. To determine the association between variables, data were Log10 transformed to normalize their distribution, and the Pearson Product Moment Correlation method was then used. A “p” value below 0.05 was considered significant. The continuous value of the mRNA expression level of IL-1α was categorized using a classification and regression tree (CART) method, considering the appearance of distant metastasis as the dependent variable. The actuarial adjusted survival free of distant metastasis was calculated using the Kaplan-Meier method. Differences in survival rates between categories defined by CART were compared by the log-rank test. A multivariate analysis was made using Cox's proportional hazard regression analysis. The dependent variable was distant metastasis-free survival, and the independent variables were location of the primary tumor (oral cavity-oropharynx *versus* larynx-hypopharynx), local extension (T1-2 *versus* T3-4), regional extension (N0 *versus* N+), type of treatment (surgery± radiotherapy *versus* radiotherapy/ chemoradiotherapy), and category of IL-1α as defined by CART. SPSS and Sigma-Plot software were used for statistical analysis.
